# Establishment of Functional Liver Spheroids From Human Hepatocyte-Derived Liver Progenitor-Like Cells for Cell Therapy

**DOI:** 10.3389/fbioe.2021.738081

**Published:** 2021-11-08

**Authors:** Wen-Ming Liu, Xu Zhou, Cai-Yang Chen, Dong-Dong Lv, Wei-Jian Huang, Yuan Peng, Hong-Ping Wu, Yi Chen, Dan Tang, Li-Na Guo, Xiu-Li Wang, Hong-Dan Zhang, Xiao-Hua Liu, Li-Qun Yang, Wei-Feng Yu, He-Xin Yan

**Affiliations:** ^1^ Department of Anesthesiology and Critical Care Medicine, Renji Hospital, Shanghai Jiaotong University School of Medicine, Shanghai, China; ^2^ Shanghai Engineering Research Center of Peri-operative Organ Support and Function Preservation, Shanghai, China; ^3^ Department of Anesthesiology, Shengli Clinical Medical College of Fujian Medical University, Fuzhou, China; ^4^ Department of Interventional Oncology, Renji Hospital, Shanghai Jiaotong University School of Medicine, Shanghai, China; ^5^ International Cooperation Laboratory on Signal Transduction, Eastern Hepatobiliary Surgery Hospital, Second Military Medical University, Shanghai, China; ^6^ College of Basic Medical Science, Dalian Medical University, Dalian, China; ^7^ Shanghai Celliver Biotechnology Co. Ltd., Shanghai, China; ^8^ Shanghai Cancer Institute, Renji Hospital, Shanghai Jiaotong University School of Medicine, Shanghai, China

**Keywords:** human hepatocytes derived liver progenitor-like cells, spheroids transplantation, acute liver failure, intraperitoneal transplantation, cell therapy, alginate microencapsulation

## Abstract

Globally, about two million people die from liver diseases every year. Liver transplantation is the only reliable therapy for severe end-stage liver disease, however, the shortage of organ donors is a huge limitation. Human hepatocytes derived liver progenitor-like cells (HepLPCs) have been reported as a novel source of liver cells for development of *in vitro* models, cell therapies, and tissue-engineering applications, but their functionality as transplantation donors is unclear. Here, a 3-dimensional (3D) co-culture system using HepLPCs and human umbilical vein endothelial cells (HUVECs) was developed. These HepLPC spheroids mimicked the cellular interactions and architecture of mature hepatocytes, as confirmed through ultrastructure morphology, gene expression profile and functional assays. HepLPCs encapsulated in alginate beads are able to mitigate liver injury in mice treated with carbon tetrachloride (CCL4), while alginate coating protects the cells from immune attack. We confirmed these phenomena due to HUVECs producing glial cell line-derived neurotrophic factor (GDNF) to promote HepLPCs maturation and enhance HepLPCs tight junction through MET phosphorylation. Our results display the efficacy and safety of the alginate microencapsulated spheroids in animal model with acute liver injury (ALF), which may suggest a new strategy for cell therapy.

## Introduction

Acute liver failure (ALF) is a rare but devastating condition with high morbidity and a mortality rate of up to 80% worldwide (Byass, 2014; Dwyer et al., 2021). Liver transplantation remains the standard therapy for medically refractory hepatic failure (Ng et al., 2018). However, graft shortages, surgical risks, and the need for lifelong immunosuppressive therapy impede greater utilization of liver transplants (Chistiakov, 2012; Jiang et al., 2019). Currently, alternative methods have been developed and used to prolong the life of patients with ALF, including hepatocyte transplantation (Zhang et al., 2018) and bridging therapies based on hybrid bio-artificial liver devices (Li et al., 2020). Recently, through chemical reprogramming, we have converted mouse and human hepatocytes into liver progenitor-like cells (HepLPCs) that could be expanded *in vitro* (Wu et al., 2017; Fu et al., 2019; Wang et al., 2019), and we established HepLPC-based bio-artificial livers (BALs) that effectively prevented acute liver failure in pigs (Li et al., 2020). However, it is still unknown whether HepLPCs can differentiate into sufficient numbers of functional hepatocytes and carry out essential liver functions for use in *in vivo* therapy.

Recently, numerous studies have reported that liver cell transplantations have failed to demonstrate improved hepatic function in clinical applications due to insufficient cell functions and restricted availability. Patients with ALF may not have enough time to wait for the cells that are produced (Bandi et al., 2019; Wu et al., 2019). It is challenging to generate sufficient numbers of long-term, high-quality, functional hepatocytes *in vitro* (Rouwkema et al., 2011; Thompson et al., 2018; Smets et al., 2019). These failures have given rise to the prevailing belief that it is problematic to mimic the complex interactions that occur among cells and tissues during organogenesis using cytokine supplement *in vitro* (Bhatia et al., 2014). An increasing number of studies have recognized that co-culture with endothelial cells could maintain the well-differentiated phenotype of cells derived from stem cells with relatively stable function (Matsumoto et al., 2001; Kidambi et al., 2009; Ding et al., 2014; Liu et al., 2016), but the mechanism is unclear. Induced pluripotent stem cells (iPSCs) play an important role in the treatment of acute liver injury in mice (Nagamoto et al., 2016; Rashidi et al., 2018; Takeishi et al., 2020), but low induction efficiency and tumorigenicity limit their wide application. iPSCs have been co-cultured with HUVECs and mesenchymal stem cells (MSCs) to form vascularized “Liver buds”. The researchers have utilized a mixed culture of iPSC-derived liver cells, HUVECs, and MSCs, for which the exact components were unknown, and the relationship between the three types of cells also was not clear (Takebe et al., 2013). Mouse liver sinusoidal endothelial cells (LSECs) can promote LPCs to improve hepatocyte function (Kim and Rajagopalan, 2010) and produce hepatobiliary organoids that can form liver-specific duct systems. The underlying mechanism of the improved hepatocyte maturation has not been explored (Yap et al., 2020). LSECs are difficult to isolate and culture currently, and this system is not suitable for large-scale cell production (Ding et al., 2014). Combining different cell types, including hepatocytes, endothelial cells, and Kupffer cells can mimic the progression of non-alcoholic fatty liver disease (NAFLD) *in vitro* (Suurmond et al., 2019), further revealing the outstanding prospects afforded by co-culture systems (Kostadinova et al., 2013; Lübberstedt et al., 2015). However, currently, no data are available concerning the differentiation mechanisms involved in 3D co-culture systems using human liver cells.

We focused on the early process involved in organogenesis, specifically, cellular interactions during organ-bud development to establish a 3D culture system from HepLPCs or a combination of HepLPCs and HUVECs (called vHepLPCs). The herpes simplex virus type 1 thymidine kinase (HSV-TK)/Ganciclovir (GCV) is a suicide gene system that can induce the reversible immortalization of human primary cells (Hossain et al., 2019). We here used a lentivirus mediated HSV-TK/GCV system in which the suicide gene expression selectively ablated proliferating HepLPCs. We further encapsulated HepLPCs/vHepLPCs spheroids in alginate beads to protect cells from attack from the immune system. These encapsulated HepLPCs/vHepLPCs spheroids successfully mitigated liver injury in mice treated with CCL4.

## Experimental Section

### Cell Culture

Cryopreserved HepLPCs were plated on a Matrigel-coated (Corning) culture dish (NEST Biotechnology) at 0.5 to 2 × 10^4^ cells/cm^2^ and cultured in transition and expansion medium (TEM) as previously described (Wu et al., 2017; Fu et al., 2019; Wang et al., 2019). HepLPCs were immortalized using lentivirus-mediated expression of simian virus 40 (SV40) large tumor (T) antigen (LT) to achieve expansion without growth arrest *in vitro*. The functionally enhanced HepLPCs were derived from selected immortalized HepLPCs by transduction using GDNF. The cells were cultured in a 37°C, 5% CO_2_ incubator, as previously described (Fu et al., 2019). The medium was changed every 3 days. The HUVEC cells were cultured in DMEM medium supplemented with 10% fetal bovine serum (FBS, Corning), penicillin (100 units/mL), and streptomycin (100 μg/ml) in a 5% CO_2_ incubator at 37°C, as previously described (Meng et al., 2017).

### Lentiviral Vector-Mediated Transduction

The pLOX-Ttag-iresTK composed of two cistrons 1) The SV40 gene encoding the large T antigen DNA (GenBank No. J02400, nt 2,691–5,163); 2)The thymidine kinase of Herpes simplex virus type 1 (HSV1-TK), downstream of the internal ribosomal entry site (IRES) of encephalomyocarditis virus (Salmon et al., 2000). This plasmid was purchased from Addgene (https://www.addgene.org). After obtained, vectors were transduced into *E. coli*. Screened clones were expanded, and plasmids were extracted and purified. Then the constructed vectors or empty vectors were co-transfected with second Generation Packaging System Mix (Abmgood, Canada) into 293T cells using calcium phosphate transfection method. After culturing transfected 293T cells in DMEM medium for 48 h, the supernatant containing lentivirus was harvested and filtered with a 0.45 μm filter (Merck Millipore, Germany).

### Ganciclovir (GCV) Treatment

Ganciclovir (GCV) was dissolved in DMSO. HepLPCs cultured *in vitro* were treated with GCV (50 μg/ml) for 2 days. For *in vivo* treatment, mice in the saline/GCV group received 50 mg/kg GCV (dissolved in 100 μL of 5% DMSO in a saline solution) intraperitoneally. For the saline group, mice received intraperitoneal injections of 100 μL of 5% DMSO in a saline solution. The GCV treatment was initiated on day two and continued over a 7-day treatment course.

### HepLPC Three-Dimensional (3D) Culture

The proliferating HepLPCs were dissociated with TrypLE Express (GIBCO), which was neutralized with pre-warmed medium, and then the cells were washed in phosphate-buffered saline (PBS). Cells were seeded at a density of 2 × 10^5^ proliferative cells per well in ultra-low attachment 6-well-plates (Corning). The culture media was based on Advance DMEM/F12 (GIBCO) supplemented with GlutaMAX-I (GIBCO), HEPES (Basal Media), Primocin (InvivoGen), 1 × B27 (GIBCO), 1.56 mM N-Acetylcysteine (Sigma, Germany), 0.5 μm A83-01 (Tocris, United State), 10 μm Y27632 (Selleck), 3 µM CHIR-99021 (SELLECK), 50 ng/ml EGF (PeproTech, United State) and 25 ng/ml HGF (PeproTech). Primary spheroids had formed by the next day. The medium was replaced every two or 3 days.

### HepLPCs/HUVECs 3D Co-culture

The proliferating HepLPCs and HUVECs were dissociated with TrypLE Express, neutralized with pre-warmed medium, and washed in PBS. 2 × 10^5^ proliferative cells (HepLPCs/HUVEC, 1:1) per well were seeded in ultra-low attachment 6-well-plates. The culture media was based on Advance DMEM/F12 supplemented with GlutaMAX-I, HEPES, Primocin, 1 × B27, 1.56 mM N-Acetylcysteine, 0.5 μm A83-01, 10 μm Y27632, 3 μm CHIR-99021, 50 ng/ml EGF and 25 ng/ml HGF. Due to the HUVEC growth conditions, we supplemented the media with 25 ng/ml vascular endothelial growth factor (VEGF, PeproTech), 10 ng/ml Fibroblast Growth Factor-10 (FGF10, PeproTech), and 1% FBS. Primary spheroids had formed by the next day. The medium was replaced every two or 3 days.

### Cell Counting Kit-8 (CCK-8) Assay

To evaluate cell proliferation, 5,000 cells per well were seeded in ultra-low attachment 96-well-plates with conditioned medium for 24 h, and then the medium was changed to medium containing 10% (v/v) CCK-8 (Dojindo) for 1 h. Cell viability was assessed every other day. Proliferation was determined by absorbance measurements at 450 nm using a multimode reader Synergy 2 (BioTek).

### LIVE/DEAD Viability Assay

The cell LIVE/DEAD viability was measured using the LIVE/DEAD® Viability/Cytotoxicity Kit *for mammalian cells* (Invitrogen).

### GDNF Overexpression

The GDNF overexpression lentivirus was purchased from Genechem (Shanghai, China). HepLPCs cells were transfected with the GDNF expression (NM_000514) construct, together with the GV492 transposase vector. Positively transposed cells were selected for 3 weeks using puromycin (2 µg/ml, Thermo Fisher Scientific).

### Transmission Electron Microscope

Cell morphology on carriers was viewed using a Transmission Electron Microscope (TEM) (Hitachi S3400N, Hitachi). Briefly, the carriers were fixed with 2.5% glutaraldehyde overnight at 4°C and then dehydrated in a series of ethanol solutions (75, 90, 95, and 100%). The resulting samples were dried and sputter-coated with gold, followed by TEM observation at a working voltage of 15 kV.

### Reverse Transcription-Polymerase Chain Reaction (RT-PCR)

RNA was isolated and purified using a RNeasy Mini Kit (Qiagen, Germany) according to the manufacturer’s instructions. The RNA concentration and purity were determined using a NanoDrop-2000 spectrophotometer (Thermo Fisher Scientific). First-strand reverse transcription was performed using 0.5–1.0 µg of RNA with PrimeScript RT Master Mix (Takara, Japan) following the manufacturer’s standard protocol. Gene expression analysis was performed using a QuantiNova SYBR Green RT-PCR Kit (Qiagen) on a Roche CFX96 Real-Time System. Gene transcription was evaluated using the ∆Ct method normalized to beta-actin (ACTB). The primer sequences and sources are listed in Supplementary Information, Table 1.

**TABLE 1:** Primers list.

Number	Name	Forward primer	Reverse primer
1	ACTB	CAT​GTA​CGT​TGC​TAT​CCA​GGC	CTC​CTT​AAT​GTC​ACG​CAC​GAT
2	ALB	TTT​ATG​CCC​CGG​AAC​TCC​TTT	AGT​CTC​TGT​TTG​GCA​GAC​GAA
3	HNF4a	GGC​CAA​GTA​CAT​CCC​AGC​TTT	CAG​CAC​CAG​CTC​GTC​AAG​G
4	CYP3A4	GTG​GGG​CTT​TTA​TGA​TGG​TCA	ACA​TCT​CCA​TAC​TGG​GCA​ATG​A
5	E-Cadherin	AAA​GGC​CCA​TTT​CCT​AAA​AAC​CT	TGC​GTT​CTC​TAT​CCA​GAG​GCT
6	ZO1	ACC​AGT​AAG​TCG​TCC​TGA​TCC	TCG​GCC​AAA​TCT​TCT​CAC​TCC
7	HGF	GCT​ATC​GGG​GTA​AAG​ACC​TAC​A	CGT​AGC​GTA​CCT​CTG​GAT​TGC
8	Ret	GTG​TCT​TCG​ATG​CAG​ACG​TG	CAT​GGT​GCG​GTT​CTC​CGA​G
9	GDNF	GGC​AGT​GCT​TCC​TAG​AAG​AGA	AAG​ACA​CAA​CCC​CGG​TTT​TTG
10	GFRA1	CCA​AAG​GGA​ACA​ACT​GCC​TG	CGG​TTG​CAG​ACA​TCG​TTG​GA
11	MMP2	GAT​ACC​CCT​TTG​ACG​GTA​AGG​A	CCT​TCT​CCC​AAG​GTC​CAT​AGC
12	VEGFA	AGG​GCA​GAA​TCA​TCA​CGA​AGT	AGG​GTC​TCG​ATT​GGA​TGG​CA
13	AFP	AGA​CTG​AAA​ACC​CTC​TTG​AAT​GC	GTC​CTC​ACT​GAG​TTG​GCA​ACA
14	KRT7	TCC​GCG​AGG​TCA​CCA​TTA​AC	GCT​CTG​TCA​ACT​CCG​TCT​CAT
15	MMP9	GGG​ACG​CAG​ACA​TCG​TCA​TC	TCG​TCA​TCG​TCG​AAA​TGG​GC

### Western Blots

Cells were lysed in RIPA Lysis and Extraction Buffer, and the protein concentrations were measured using a Pierce™ BCA Protein Assay Kit (Both from Thermo Fisher Scientific). Proteins were subjected to electrophoresis on 8–10% Bis-Tris protein gels and transferred to nitrocellulose membranes (GE Healthcare), which were incubated with the primary antibodies, followed by HRP-conjugated secondary antibodies. The antibodies that were used are listed in Supplementary Information, Table 2. The fluorescence density on the nitrocellulose membranes was measured using a ChemiDoc™ XRS + system (BIO-RAD).

**TABLE 2:** Antibodies list.

Number	Antibody	Company	Code number
1	GAPDH	Cell signaling technology	8,884
2	HNF4a	Cell signaling technology	3,113
3	Albumin	Proteintech	16475-1-AP
4	Albumin	BETHYL	A80-229A
5	CD31	Cell signaling technology	3,528
6	CYP3A4	Proteintech	18227-1-AP
7	Met	Cell signaling technology	8,198
8	Phospho-Met (Tyr1234/1,235)	Cell signaling technology	3,077
9	ZO2	Cell signaling technology	2,847
10	Ret	Cell signaling technology	14556
11	GDNF	Abclonal	A14734
12	AAT	BETHYL	A80-122A-20
13	ZO-1	Cell signaling technology	13663
14	Ki-67 (D2H10)	Cell signaling technology	9,027
15	E-cadherin	Cell signaling technology	14472
16	MRP	Abcam	ab121287

### Spheroid Immunofluorescence (IF)

The spheroids were fixed with formalin or 4% paraformaldehyde (PFA), then blocked and permeabilized in PBS containing 5% goat serum and 0.1% Triton-X-100. For IF, the spheroids were incubated with primary antibodies as follows: anti-ALB, anti-AAT (Bethyl Laboratories, United State), anti-HNF4a, anti-KRT19, anti-E-cadherin (Cell Signaling Technology, United State), anti-CYP3A4 (Proteintech, United State) and anti-MRP (Abcam, England). The spheroids were incubated in appropriate secondary antibodies for 1h at room temperature in the dark. The secondary antibodies used for immunofluorescence were as follows: Alexa fluor 594 donkey anti-goat IgG, Alexa fluor 488 donkey anti-mouse IgG, Alexa fluor 488 donkey anti-rabbit IgG, Alexa fluor 594 donkey anti-rabbit IgG, and Alexa fluor 594 donkey anti-mouse IgG (Life Technology). The nuclei were stained with DAPI (Sigma). We used an Olympus FV3000 confocal microscope to obtain the image data.

### Tissue Immunohistochemistry (IHC)

Tissue paraffin embedding was conducted by using a previously described method (Gao et al., 2014). Paraffin-embedded tissues or organoids were cut into 4 μm sections on a microtome, mounted on glass microscope slides, and stored at room temperature. Paraffin sections were deparaffinized and underwent antigen retrieval in sodium citrate buffer (pH 6.0) in a steamer for 18 min. The sections were blocked and permeabilized in PBS with 5% goat serum and 0.1% Triton-X-100. Then the sections were incubated with primary antibodies in 2% Bovine serum albumin (BSA) overnight at 4°C. The next day, samples were incubated with appropriate secondary antibodies (Zsbio, China), following the manufacturer’s instructions.

### ALB and HGF ELISA

Human ALB was measured using the Human Albumin Quantification kit (Bethyl Laboratory). HGF was measured using the Human HGF ELISA kit (MULTI SCIENCES).

### Periodic Acid-Schiff (PAS), Oil Red, and Indocyanine Green (ICG) Staining

The PAS staining system was purchased from Sigma-Aldrich and conducted according to the manufacturer’s instructions. Oil red staining was carried out following the user manual (Sigma-Aldrich). The ICG uptake assay was performed. Briefly, the spheroids were cultured with 1 mg/ml ICG (Sigma-Aldrich) and incubated at 37°C for 1 h, washed three times in culture medium, and images were captured with the Olympus DP80 microscope.

### Transplantation

All animal experiments in the current study, including the experimental procedures, sample isolation, and animal care, were approved by and were carried out in accordance with the guidelines and regulations of the Shanghai Model Organisms Center Inc., Institutional Animal Care and Use Committee. For acute liver injury, previous studies demonstrated that CCL4 (Sinopharm Chemical Reagent, China) could be utilized to establish a liver injury model using NSG mice (Varela-Moreiras et al., 1995). 200 µL of a 10% CCL4 solution was administered intraperitoneally to eight-week-old NSG mice (Shanghai Model Organisms Center, China). 24 h after CCL4 administration, encapsulated HepLPCs, or encapsulated vHepLPCs were transplanted into the livers of NSG mice using intraperitoneal injection. Besides, we also transplanted HepLPCs, or vHepLPCs into the livers of the NSG mice via intrasplenic injection. Blood samples were collected from surviving mice in a 24 h interval. Seven days after transplantation, tissues from the surviving animals were prepared for paraffin sections. The sections were deparaffinized and stained with hematoxylin and eosin (H&E) and for Ki67, as described previously (Huang et al., 2014). Mice were selected randomly for liver injury or used as control mice in all experiments.

### Statistics

All data are presented as means ± SEM. All statistical analyses were performed using GraphPad Prism 7. For comparison between two mean values, a two-tailed unpaired t-test was used to calculate statistical significance. The one-way ANOVA was used with Dunnett correction for multiple comparisons of multiple values to a single value or Tukey correction for multiple comparisons of multiple values to each other. A *p*-value < 0.05 was considered statistically significant.

## Results

### Establishment of vHepLPC Spheroids Using a 3D Co-culture System

We previously identified small-molecule–based culture conditions that allowed for the conversion of mouse and human hepatocytes to convert into HepLPCs *in vitro* (Wu et al., 2017; Fu et al., 2019; Wang et al., 2019). To confirm the safety of these cells, we tested the effect of HSV-TK system in HepLPCs. We observed that 48 h GCV treatment resulted in cell death in a dose-dependent manner (Supplementary Figure S1A). HepLPCs that were HSV-TK positive died after GCV treatment (Supplementary Figure S1B). The PI positive cells were significantly increased after GCV treatment (Supplementary Figure S1C). To further verify the safety of these cells *in vivo*, we inoculated HepLPCs at a high density (5 × 10^6^) subcutaneously in immunodeficient mice. After that GCV treatment was given and tissue samples were obtained 7 days later. We found that HepLPCs transduced with GFP were considerably reduced following GCV treatment (Supplementary Figure S1E). TUNEL staining revealed that apoptotic cells increased substantially in GCV-treated mice (Supplementary Figure S1G). These results showed that GCV resulted in considerable death of HSV-TK positive HepLPC cells in a dose-dependent manner.

**FIGURE 1:**
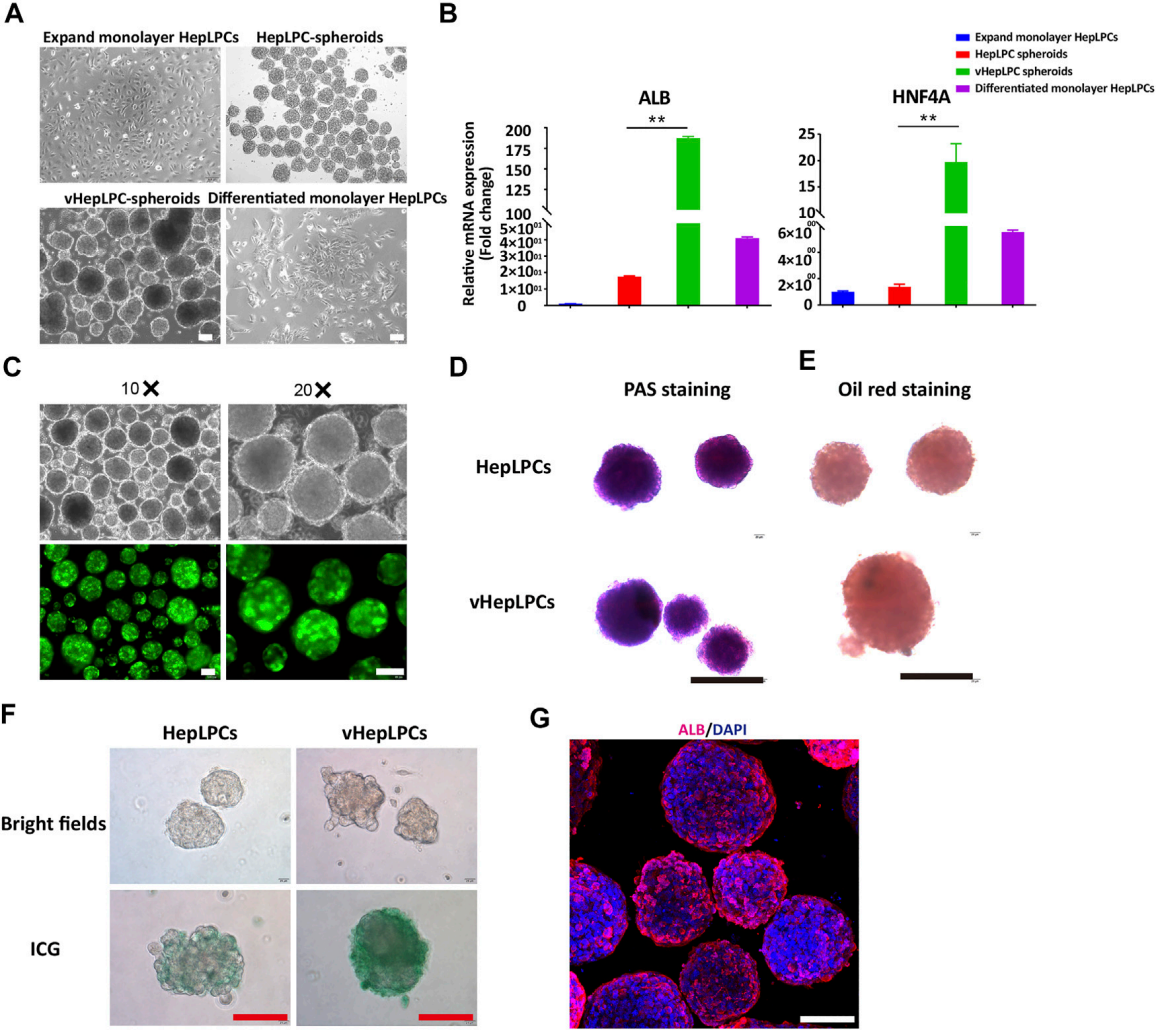
Establishment of a co-culture system using HepLPCs and HUVECs. **(A)** Representative bright-field images of HepLPCs in expandable monolayer HepLPCs, HepLPCs spheroids, vHepLPC spheroids, and differentiated monolayer HepLPCs. **(B)** Gene expression was analyzed by qRT-PCR. Hepatic functional markers (ALB, HNF4A) were detected in four groups. Data are normalized to the expandable monolayer HepLPCs group. **(C)** Fluorescence morphology of HepLPC spheroids on carriers. **(D–F)** Periodic acid-Schiff (PAS) staining, oil red staining, and ICG staining of HepLPCs (upper panel) and vHepLPC spheroids (lower panel). **(G)** The expression of ALB was determined by immunofluorescent staining in vHepLPCs. Nuclei were counterstained with DAPI. Data are shown as mean ± SEM. **p < 0.01. Scale bars, 100 µm.

To investigate the influence of HUVEC on HepLPC maturation, we analyzed the morphologies and gene expression levels of 2D HepLPCs, 3D HepLPCs spheroids, 3D HepLPC + HUVECs (1:1 mix) spheroids (vHepLPCs), and 2D differentiated HepLPCs (Figures 1A,Figures 1B). We observed that human HepLPCs cultured with HUVECs in ultra-low attachment 6-well-plates could self-organize into macroscopically visible three-dimensional spheroids within 24 h after seeding. The expression of *ALB* and *HNF4A* in the vHepLPCs was significantly higher than that in the other groups (Figure 1B). Co-culture for 5 days efficiently maintained the high levels of mature hepatocyte genes, compared with 2D cultures. These results are consistent with the conclusions obtained by Dae-Soo Kim using the liver-specific gene expression panel (LiGEP) algorithm (Kim et al., 2017). We developed a defined medium that prevented vHepLPCs spheroids from cell death with little Alpha-fetoprotein (AFP) expression (Supplementary Figure S2). GFP images of HepLPC spheroids revealed that they exhibited intact morphology (Figure 1C). PAS staining, oil red staining, and ICG uptake of HepLPC spheroids (upper panel) and vHepLPC spheroids (lower panel) demonstrated that both cell types partially reverted to a status that was similar to mature hepatocytes (Figures 1D–F). The majority of vHepLPC spheroids were positive for the mature hepatocyte marker, albumin (Figure 1G). Moreover, we found that co-culture with HUVECs was the most effective in promoting hepatic maturation than HGF, VEGF, or vHepLPC-conditioned medium alone (Supplementary Figure S3). Therefore, we established a HepLPC + HUVECs (1:1) co-culture system, which was also cost-effective and non-tumorigenic (Supplementary Figure S2).

### Functional Characteristics of vHepLPC Spheroids *in vitro*

**FIGURE 2:**
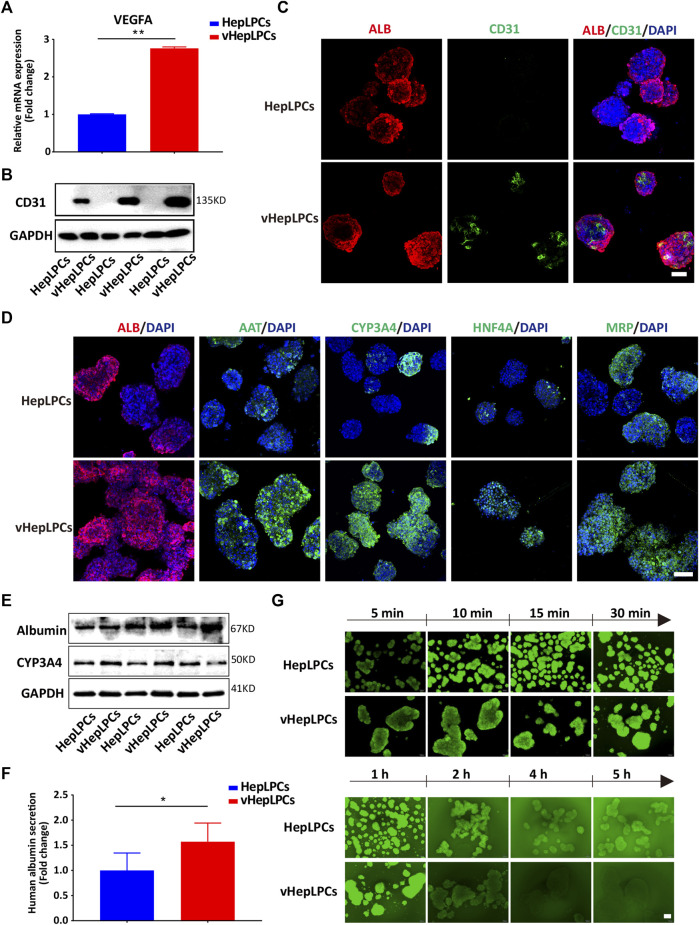
Co-culture of HepLPCs and HUVECs promoted HepLPCs maturation. **(A)** VEGFA expression was measured by qRT-PCR. Data are normalized to HepLPCs. **(B)** Western blot analysis of the expression of CD31 (*n* = 3 per group). **(C)** The expression of ALB and CD31 in HepLPCs and vHepLPCs was determined by co-immunofluorescent staining. Nuclei were counterstained with DAPI. **(D)** Immunofluorescent staining analyses of the expression of mature hepatic markers after co-culture with HUVECs, ALB, AAT, CYP3A4, HNF4A and MRP. Nuclei were counterstained with DAPI. **(E)** Western blot analysis of the expression of ALB and CYP3A4 in two groups (*n* = 3 per group). **(F)** Quantitative analysis of ALB secretion in supernatants. Data are normalized to HepLPCs (*n* = 3 per group). **(G)** Time-lapse imaging of microvilli network in HepLPCs and vHepLPCs at 5 min to 5 h of incubation with CDFDA (1 μM). Fluorescence images show both cell types can transport CDFDA, but vHepLPCs exhibited faster transport efficiency than HepLPCs. Data are presented as means ± SEM. *p < 0.05, **p < 0.01. Scale bars, 100 µm.

Next, we examined the mRNA and protein levels of vHepLPC spheroids. The expression of endothelial cell markers (VEGFA and CD31) was detected in the vHepLPCs (Figures 2A–C). The hepatic gene expressions, including ALB, AAT, CYP3A4, HNF4A, and MRP (multidrug resistance-associated protein) were strikingly improved in vHepLPCs. The cell spheroids showed striking consistency in gene expression (Figure 2D). The expression of CYP3A4 was visibly increased after rifampicin treatment (Supplementary Figure S4). Different groups of proteins further confirmed these results (Figure 2E). We also detected ALB secretion in the supernatant. The vHepLPCs secreted considerably more albumin and displayed more robust function (Figure 2F). Interestingly, we observed that HepLPC and vHepLPC spheroids both transported 5 (6)-carboxy-2',7'-dichlorofluorescein diacetate (CDFDA), but the vHepLPCs displayed faster transport efficiency than the HepLPCs (Figure 2G). This implied that the vHepLPCs had better metabolic capacity. In conclusion, HepLPCs and HUVEC co-culture mimicked the cellular interactions and architecture of mature hepatocytes and significantly improved the function of HepLPCs. Furthermore, the co-culture of HepLPCs and HUVEC led to a remarkable spheroid homogeneity (Figure 2D), suggesting that epithelial and endothelial cell-cell communication is contributable to hepatocyte maturation.

### VHepLPC Spheroids Ameliorated Liver Injury *in vivo*

**FIGURE 3:**
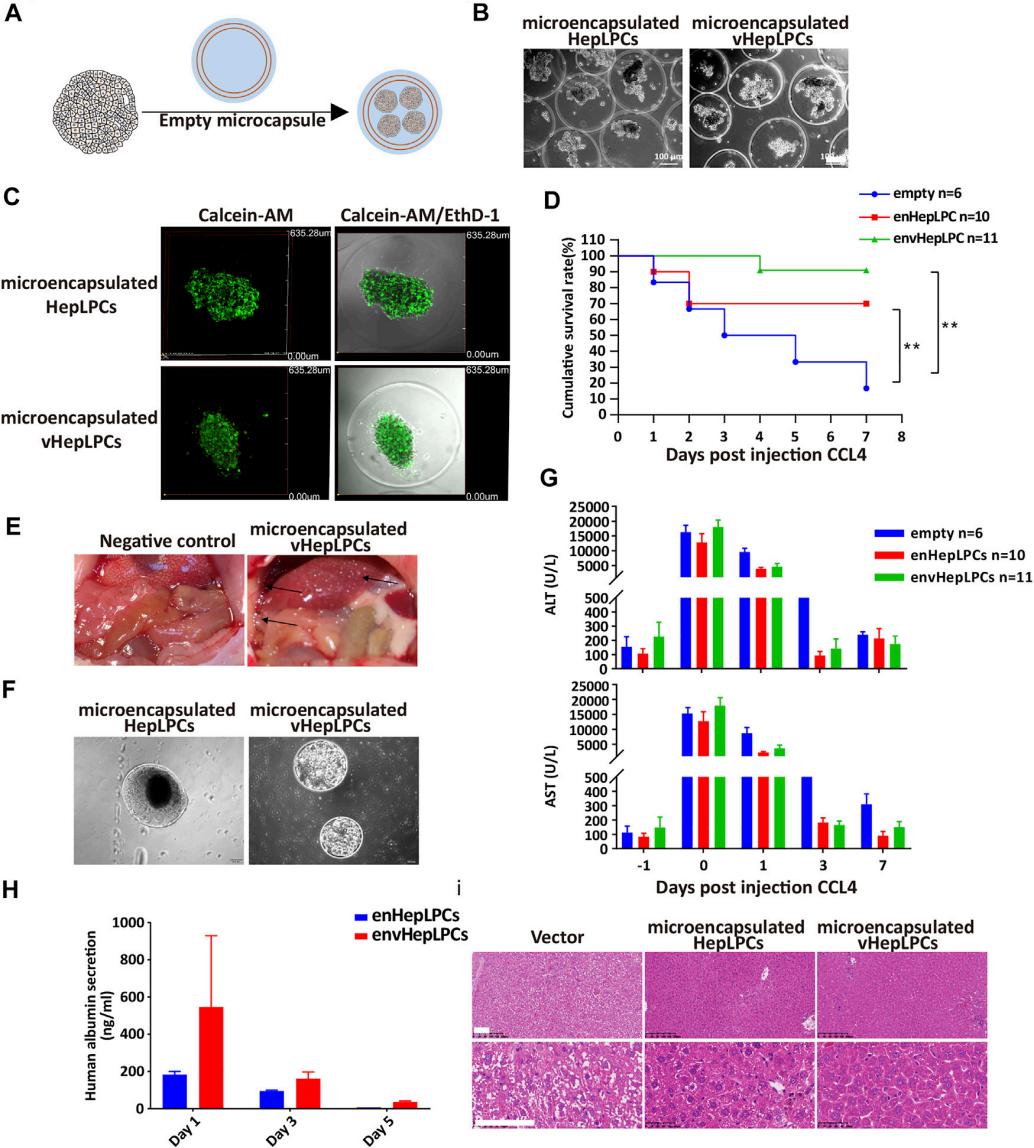
Alginate microencapsulated vHepLPC spheroids significantly improved the survival rate of mice with liver injury. **(A)** Alginate microcapsule coating protocol diagram. **(B)** Bright-field image of coated spheroids. **(C)** Cell viability test after microencapsulation. **(D)** Survival curve for acute liver injury mice with microencapsulated HepLPC spheroids (*n* = 10), vHepLPC spheroids (*n* = 11), or empty (*n* = 6) transplantation. **(E)** The microcapsule particles were enriched around the liver after transplantation. **(F)** Microcapsules recovered from the mouse abdominal cavity. **(G)** Serum levels of ALT and AST in CCL4-treated mice before and after transplantation (days one through seven) of microencapsulated spheroids. **(H)** Serum levels of human albumin secretion in CCL4-treated mice. **(I)** H&E staining 7 days after transplantation of alginate microencapsulated spheroids. The liver tissue after transplantation was significantly closer to normal tissue morphology. Data are presented as means ± SEM. **p < 0.01. Scale bars, 100 µm.

To examine the *in vivo* function of vHepLPC spheroids, we embedded spheroids in alginate microbeads, followed by transplantation into NSG mice by intraperitoneal injection (Figure 3A). Encapsulation of HepLPCs or vHepLPCs maintained cell viability (Figures 3B,C). The alginate-microencapsulated HepLPC spheroids significantly improved the survival rate of mice with CCL4-induced liver injury. In the first 3 days, the mortality rate in the control group was 50%, the mortality rate in the enHepLPCs group was 30%, and the mortality rate in the envHepLPCs group was 0%. Within 7 days, the mortality rate of the control group was 86%, the mortality rate of enHepLPCs was 30%, and the mortality rate of envHepLPCs was 9%. (Figure 3D). For 1 month, the mortality rate of the three groups has not changed compared with that of 7 days. The safety and efficacy of hepatocyte microbeads transplanted intraperitoneally have been noted after intraperitoneal transplantation of HepLPC microbeads while the microbeads were able to distribute freely within the peritoneal cavity. Many microbeads were seen attached to the omentum (black arrow), and no inflammation or fibrosis was observed (Figure 3E). Representative images of microbeads retrieved at 48 h after liver transplantation are seen in Figure 3F and the cell morphology was still complete. Encapsulation of cells with alginate microcapsules can maintain cell viability by resisting high shear forces and inhibiting the immune response due to the effects of the physical isolation (Yang et al., 2019). Serum levels of alanine aminotransferase (ALT) and aspartate aminotransferase (AST) in CCL4-treated mice were assessed before (−1) and after (days one to seven) transplantation of microencapsulated spheroids. The ALT/AST of the experimental group decreased significantly the next day (Figure 3G). The levels of albumin synthesis from human vHepLPC microbeads on days three and five were significantly lower than on the first day after transplantation (Figure 3H). H&E staining was carried out 7 days after transplantation of alginate microencapsulated spheroids. The liver tissue after transplantation significantly resembled normal liver tissue morphology (Figure 3I). At the same time, we have confirmed these results via intrasplenic transplantation experiments. The survival rate for the vHepLPC transplanted mice was significantly higher than for the HepLPC transplanted or control mice. The H&E staining revealed acute hepatic failure that was often accompanied by the loss of numerous parenchymal cells. However, there were considerably more Ki67 positive cells in the damaged tissue after transplantation of vHepLPC spheroids (Supplementary Figure S5).

### Structural Characteristics of vHepLPC Spheroids *in vitro*

**FIGURE 4:**
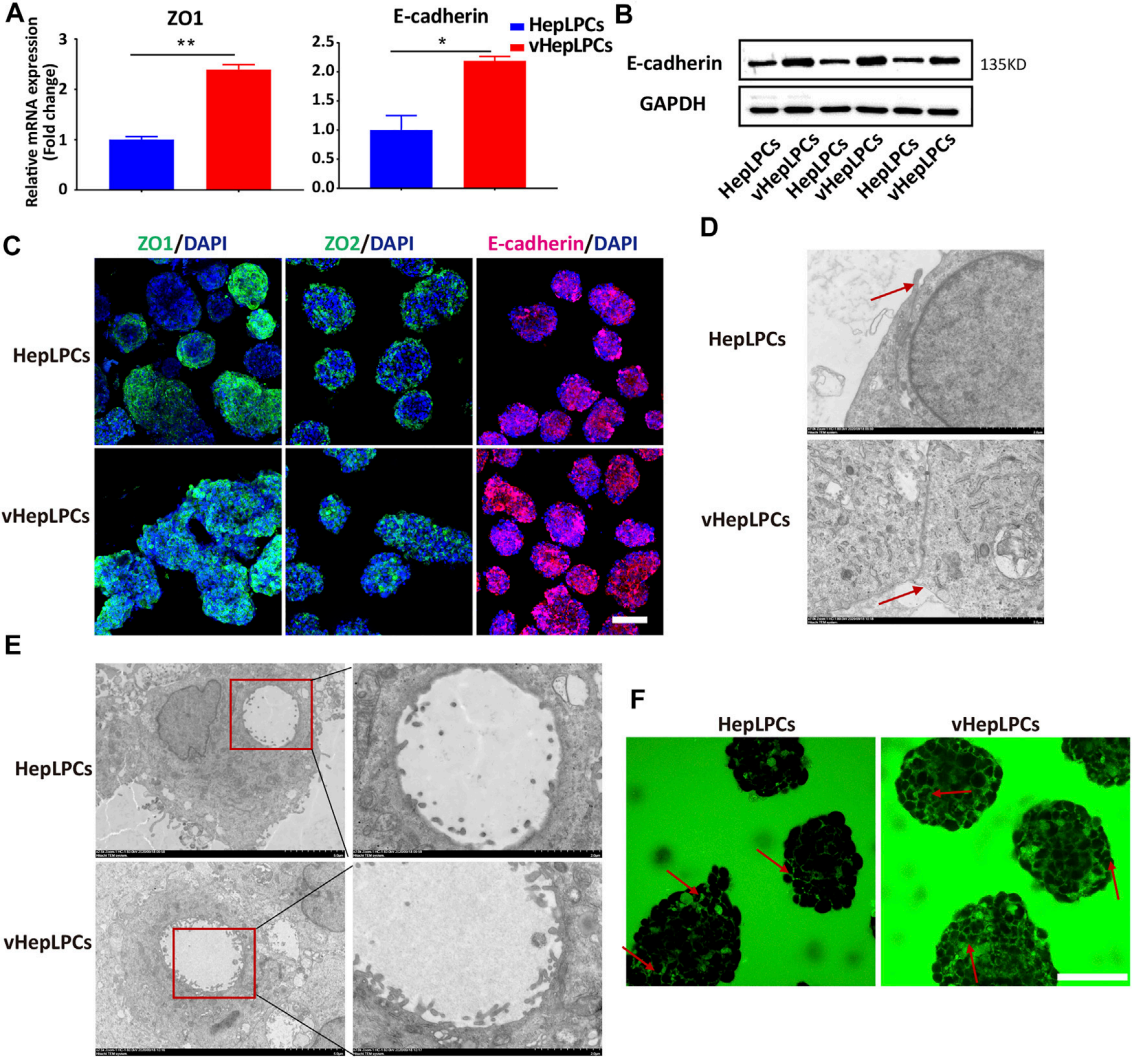
Co-culture of HepLPCs and HUVECs enhanced cell-cell tight junctions. **(A)**Gene expression was analyzed by qRT-PCR. Tight adhesion markers (ZO1, E-cadherin) were assessed. Data are normalized to HepLPCs. **(B)** Western blot analysis of the expression of E-cadherin in two groups (*n* = 3). **(C)** Immunofluorescent staining analyses of the expression of the tight junction markers, ZO1, ZO2, and E-cadherin, in HepLPCs and vHepLPCs. Nuclei were counterstained with DAPI. **(D)** Transmission electron microscopy results showed tight junctions between cells. vHepLPCs exhibited more complete tight junctions between cells, as indicated by the red arrow. **(E)** Representative TEM images. Electron microscopic analysis showed that the abundance and height of microvilli in vHepLPCs were significantly higher than in HepLPCs, suggesting that these structures are morphologically equivalent to villi that form in the bile canaliculi. **(F)** Live-imaging of CDF flow pathway from HepLPCs and vHepLPC spheroids. Red arrow pointed that the bile canaliculi network was formed by continuous tight junctions between polarized hepatocytes in vHepLPCs in magnificated pictures. Data are presented as means ± SEM. *p < 0.05, **p < 0.01. Scale bars, 100 µm.

Structure determines function, the functional improvement of vHepLPC spheroids must be accompanied by structural changes. Thus, we assessed the structural characteristics of our spheroids. We observed that tight junctions between cells were distinctly enhanced after co-culture (Figures 4A–C). We verified that the spheroids expressed tight junction proteins, ZO1, ZO2, and other associated transmembrane proteins, such as E-cadherin. Their expression was more consistent in vHepLPCs (Figure 4C). Transmission electron microscopy results demonstrated that the tight junctions between cells were more complete in vHepLPCs (Figure 4D). Troglitazone and its derivative troglitazone sulfate have been reported to induce cholestasis by inhibiting the activity of bile acid transporters (Ramli et al., 2020). Based on these reports, we treated vHepLPC spheroids with troglitazone. Time-lapse imaging showed a substantial increase in cytoplasmic CDF in the cells of the vHepLPC spheroids after troglitazone exposure (Supplementary Figure S6A). Consistent with reports in the literature, CDFDA transport was attenuated by destroying hepatocytes and had no significant effect on cholangiocytes (Supplementary Figure S6B). Also, we examined the structures formed in the spheroids at high resolution using TEM and observed microvilli at the junctions of the HepLPC/vHepLPC spheroids. The height and abundance of microvilli increased prominently in the vHepLPCs (Figure 4E). However, the formation was likely dependent on the interactions and communication of the HepLPCs and HUVECs. We attempted to capture the flow of CDF from the microtubule network into the cyst structures with time-lapse imaging. We observed that a continuous flow pathway was formed in vHepLPCs (Figure 4F). Overall, these data further demonstrated that our spheroids possessed the capacity to extend microvilli. Incorporating HUVECs promoted the maturation and function of HepLPCs, further enhancing intercellular tight junctions.

### HUVECs-Produced GDNF Promoted HepLPC Maturation

**FIGURE 5:**
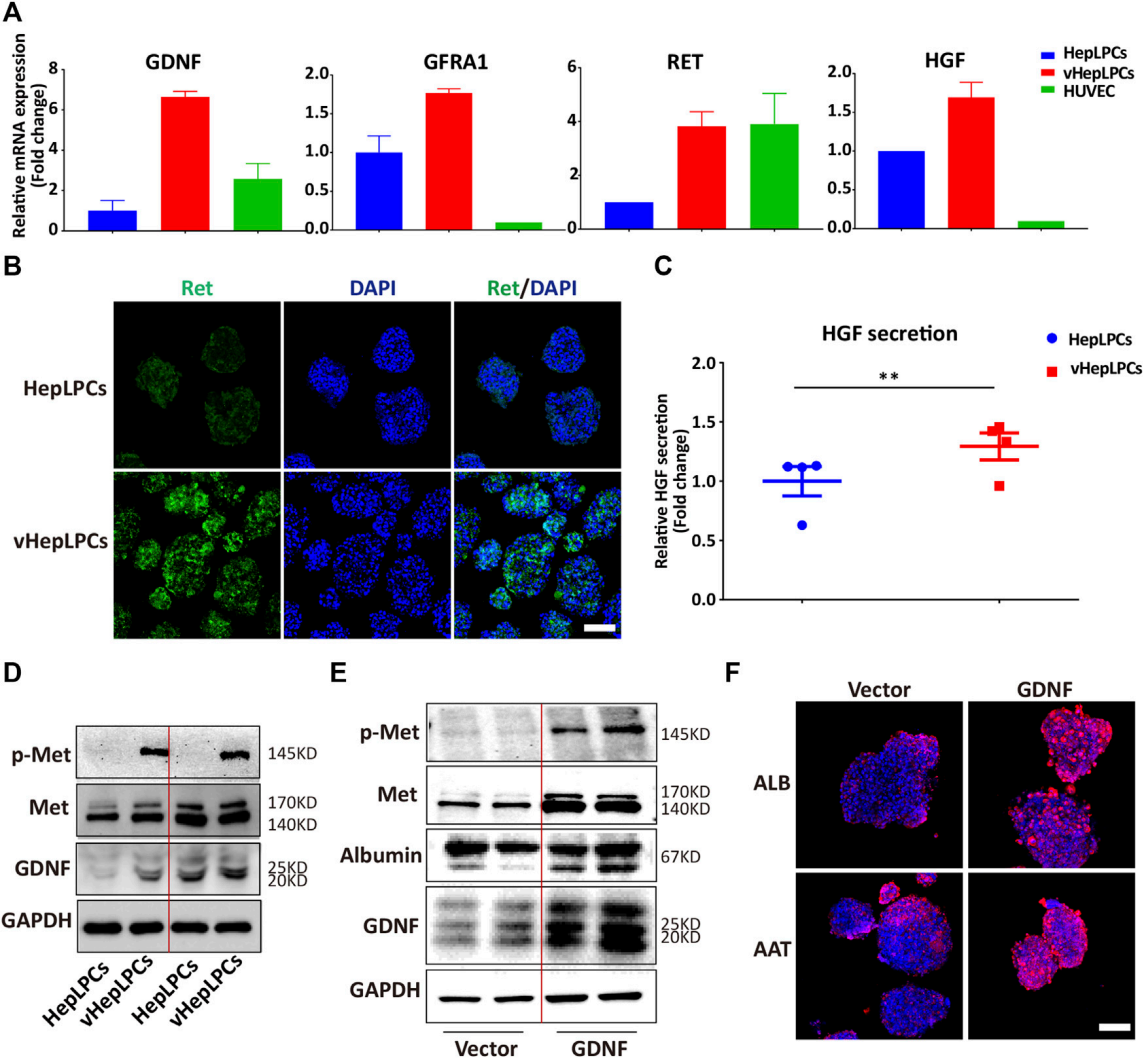
HUVECs produced GDNF that promoted HepLPC maturation by phosphorylation of Met. **(A)** Gene expression was analyzed by qRT-PCR. GDNF, GDNF receptors (Ret, GARF1), and HGF were analyzed. Data are normalized to HepLPCs. **(B)** Immunofluorescence confirmed that the expression of the GDNF receptor, Ret, increased after co-culture. **(C)** Quantitative analysis of HGF secretion in supernatants. Data are normalized to HepLPCs (*n* = 4 per group). **(D)** The expression of different batches of Met, p-Met, and GDNF after co-culture was assessed using western blots (*n* = 3 per group). **(E)** Western blots were used to detect GNDF, Met, and p-Met in HepLPCs and HepLPCs that overexpressed GDNF (*n* = 3 per group). **(F)** Immunofluorescent staining analyses of the expression of hepatic function markers (ALB, AAT) in HepLPCs and HepLPCs that overexpressed GDNF. Nuclei were counterstained with DAPI.

We showed that HepLPC and HUVEC co-culture promoted the maturation of HepLPCs, and enhanced cell-cell tight junctions. However, the mechanism was not clear. We screened the factors secreted by HUVECs, including VEGF, matrix metallopeptidases (MMPs), and GDNF(Miya et al., 2011; Nakasatomi et al., 2019). Unexpectedly, we found that GDNF expression was increased significantly in vHepLPCs (Supplementary Figure S7). As reported that bone marrow stromal cells with high functionality was correlated with elevated levels of GDNF (Ma et al., 2016). Previous studies on GDNF have often focused on nerves and kidneys (Ma et al., 2016; Popsueva et al., 2003; Wang et al., 2018). The potential involvement of GDNF in liver differentiation has not been reported previously (Fiore et al., 2009). The receptor complex for GDNF consists of the rearranged in transfection (Ret) receptor tyrosine kinase and glycosylphosphatidylinositol (GPI)-linked GDNF family receptor1 (GFRa1) (Airaksinen et al., 1999). We proved that the expression of the mRNA levels for the GDNF receptors, *Ret* and *GFRA1,* increased substantially (Figure 5A). Concomitantly, Ret immunofluorescence also was enhanced (Figure 5B). The literature has documented that the HGF binds to the tyrosine kinase receptor (Met) and then performs functions as a multi-functional cytokine (Van Belle et al., 1998). Not surprisingly, HGF secretion was considerably higher than in controls (Figure 5C). We also observed that Met was activated in vHepLPCs (Figure 5D). To assess the relationship between GDNF and the maturation of HepLPCs, we constructed stable lines of HepLPCs that overexpressed GDNF. The GDNF expression was upregulated after HepLPCs GDNF overexpression, and Met phosphorylation was induced (Figure 5E). Concomitantly, the increased expression of ALB and AAT proteins was verified using immunofluorescent staining, demonstrating that over 90% of HepLPCs were positive for both ALB and AAT after GDNF overexpression (Figure 5F). These results suggested that HUVECs-produced GDNF acts through activation of Met to promote HepLPC maturation and form advanced structure.

## Discussion

The transplantation of progenitor cells to the liver as replacement therapy to treat chronic, genetic, and end-stage liver diseases has engendered intense interest in the field of hepatic stem cell therapy (Miyajima et al., 2014; Fu et al., 2019; Ko et al., 2020). The generation of functional and proliferating hepatocytes is a necessarily required for liver tissue engineering. A bio-artificial liver support system could serve as a potential complementary therapy for the replacement of liver cells or transplantation in patients with ALF (Shi et al., 2016; Li et al., 2020). Due to the cell resource and appropriate bioreactor of *in vitro* bio-artificial liver device, the concept of establishing an *in vivo* cell-based BAL system has become increasingly urgent. In this study, vHepLPCs were used to develop a safe and feasible *in vivo* bio-artificial liver. Overall, the data demonstrated that our protocol efficiently generated a support system that satisfied all four criteria that defined spheroids, including 1) the safety of the cell resources, 2) development of liver function, 3) therapeutic efficacy after transplantation, and 4) elaboration of its differentiation mechanism.

First, the clinical safety of this support system focused on the resources of the hepatocytes. Recently, four main cell sources have been studied concerning their probability as donor sources for hepatic stem cell transplantation (Li et al., 2020). Human primary hepatocytes (PHHs) are the most appropriate cell source for cell transplantation. However, due to the shortage of organ donors, the current model of PHHs culture is not suitable for large-scale amplification (Xiang et al., 2019). Hepatoma cell lines, such as HepG2 cells or others, are capable of spontaneous expansion and secretion of albumin *in vitro*, but, the risk of potential tumorigenicity cannot be ignored (Tang et al., 2008). Moreover, these cells lack the ability to synthesize urea through the action of the urea cycle. Although porcine hepatocytes are physiologically closest to human hepatocytes, their xenogeneic origin poses two major problems, which are the risk of xenogeneic infections and immune reactions (Zhang et al., 2020). Human-induced hepatocytes (hiHeps) that have been reprogrammed from iPSCs(Cheng et al., 2012), and human embryonic stem cells (hESCs) (Feng et al., 2020) have the potential of carrying out detoxification and metabolic functions. However, potential safety risks that are inherent in the complicated gene editing experiments and therapeutic applications cannot be avoided. HepLPCs show the huge possibilities for developmental biology, and cell therapy, which as a suitable source becomes available for transplantation.

Second, the potential benefits of this support system focused on hepatocyte function. Our system not only recapitulated but improved the function of the HepLPCs. The morphological and functional characteristics of cells in co-culture systems were evaluated in comparison with homotypic cultures and traditional systems. Previous studies mainly relied on co-cultivation that the composition of the medium is complicated and not well-defined. The system developed in this study was that the composition of the medium was clear and the time needed for culture was often less than 7 days. Thus, the costs for culture were reduced, the culture period was shortened, and the complete protocol was simplified. Providing an adequate foundation for cell engineering culture is an essential factor. Another important outcome of our study was that numerous, highly functional hepatocytes were obtained that could meet the cell numbers required for clinical transplantation. Furthermore, although more detailed analyses are required, our study reinforced the mechanism of LPC differentiation and the application of LPC. We are hopeful that this method will allow additional study of human liver development and modeling of liver diseases.

Third, the safety of the clinical applications of this support system focused on avoiding clearance by the host immune response. Cell delivery in cell therapy is typically challenged by a low cell survival rate and immunological rejection during the injection and circulation of the cells (Li et al., 2020). Injection of cells into the portal venous system or spleen carries a potential risk of bleeding in coagulopathic patients and the need for long-term immunosuppression. Encapsulate the spheroids in alginate, which is a bio-inert material that protects the hepatocytes from the host immune system while allowing substrates and proteins to pass freely through the material. Hepatocytes encapsulated in alginate microcapsules are attractive sources of cell-based therapies to treat ALF (Ng et al., 2018). The HSV-TK/GCV gene therapy system has been implemented extensively, along with the promising suicide gene/prodrug system (Hossain et al., 2019). The effectiveness depends on producing sufficient transfection efficiency of the HSV-TK genes into the HepLPCs. Also, the phosphorylated GCV is capable of moving through intercellular tight junctions to spread its cytotoxicity to adjacent HepLPCs that do not express HSV-TK. Based on these clinical features, the remaining HepLPCs can be completely and safely removed following liver cell transplantation. We have successfully established vHepLPC spheroids that are suitable for transplantation and should be useful in a variety of applications.

Fourth, our system also revealed aspects of the mechanisms underlying cell to cell interactions. In this study, we adopted HUVECs only, as HUVECs secreted VEGF, MMPs, GDNF, and others (Fiore et al., 2009). We determined that vHepLPCs produced more MMPs, and these matrix components appeared to recapitulate the three-dimensional microenvironments that promote cells to interact and communicate with each other (Kanninen et al., 2016). Indeed, we confirmed that the tight junctions between cells were enhanced after co-culture based on the observed morphology, mRNA, and protein levels. It is reported that GDNF is related to branching and differentiation (Pepicelli et al., 1997; Popsueva et al., 2003; Shakya et al., 2005). We found that GDNF expression increased after co-culture and Met was phosphorylated, leading to the improved function of the HepLPCs. These results suggest that GDNF contributed to hepatocyte–endothelial cell–cell communication. Collectively, our study might also provide a potential model for investigating the mechanisms underlying cellular differentiation.

## Conclusion

In conclusion, we developed a safe and feasible *in vivo* bio-artificial liver, these alginate microencapsulated spheroids exhibited liver function upon transplantation and prevented liver failure in mice. Meanwhile we revealed HUVECs produced GDNF to promote hepLPCs maturation and enhance hepLPCs tight junction, and these phenomena were related to MET phosphorylation.

## Data Availability

The original contributions presented in the study are included in the article/Supplementary Material, further inquiries can be directed to the corresponding authors.

## Supplementary Material

Supplementary Figure S1 The safety and efficacy of HepLPCs expressing HSV-TK. **(A)** GCV had a significant inhibitory effect on HepLPCs. Data are normalized to the DMSO group. **(B)** Cells appeared died after GCV treatment. **(C)** The Calcein-AM/PI assay was performed after GCV treated for 48h. **(D)** The PI positive cells were calculated from three independent experiments. Data are normalized to the DMSO group. **(E)** Cells were inoculated subcutaneously in immunodeficient mice, *n*=5. **(F)** The cell viability (GFP positive cells) was calculated from three independent experiments. Data are normalized to the DMSO group. **(G)** The TUNEL assay was performed after GCV treatment. **(H)** The apoptotic cells (TUNEL staining positive cells) were calculated from three independent experiments. Data are normalized to the DMSO group. Data are represented as means ± SEM. **p <* 0.05 and ***p <* 0.01. Scale bars: 100 μm.

Supplementary Figure S2 vHepLPCs had a higher cell viability than HepLPC spheroids. **(A)** Assessment of cell viability using Calcein-AM/PI staining. **(B)** qRT-PCR analysis of the expression of AFP. Data are normalized to the HepLPC group. Data are represented as means ± SEM. **p* < 0.05. Scale bar, 100 μm.

Supplementary Figure S3 HUVECs played an important role in co-culture system. **(A)** Bright-field images of HepLPCs in different culture conditions. **(B)** qRT-PCR analysis of hepatic markers in different culture conditions. Data are normalized to the HepLPC group. Data are represented as means ± SEM. **p* < 0.05 and ***p* < 0.01. Scale bar, 100 µm.

Supplementary Figure S4 Rifampicin enhanced CYP3A4 expression. **(A)** IF analysis of CYP3A4 expression in HepLPCs after Rifampicin treatment for 72 h. bar, Scale bar, 100 µm.

Supplementary Figure S5 Transplantation of vHepLPC spheroids promoted functional recovery in mice with acute liver injury. **(A)** Survival curve for acute liver injury mice with HepLPCs (*n*=11), vHepLPCs (*n*=10), or without (*n*=11) spheroid transplantation. **(B)** Serum levels of ALT and AST in CCL4-treated mice before (−1) and after transplantation (days one through seven) of spheroids. **(C)** H&E and immunohistochemistry staining for Ki67 after transplantation of human spheroids on day two and day thirty. Data are presented as means ± SEM. Scale bars, 100 µm.

Supplementary Figure S6 Troglitazone inhibited CDFDA excretion by destroying hepatocytes. **(A)** Live-imaging of CDF in vHepLPCs incubated with troglitazone (80 μM) for 6 h. **(B)** ALB and KRT7 were analyzed by qRT-PCR. Data are normalized to the DMSO group. Data are presented as means ± SEM. **p* < 0.05. Scale bar, 100 µm.

Supplementary Figure S7 Screening HUVECs-derived factors. **(A)** HUVECs were screened for derived cell factors using qRT-PCR. Data are presented as means ± SEM. **p* < 0.05.

## Glossary

3D: 3-dimensional
4% PFA: 4% paraformaldehyde
AAT: Alpha-antitrypsin
ACTB: Beta-actin
AFP: Alpha-fetoprotein
ALB: Albumin
ALF: Acute liver failure
ALT: Alanine aminotransferase
AST: Aspartate aminotransferase
BAL: Bio-artificial livers
BSA: Bovine serum albumin
CCK8: Cell counting kit-8
CCL4: Carbon tetrachloride
CDFDA: 5 (6)-carboxy-2',7'-dichlorofluorescein diacetate
CYP3A4: Cytochrome P450 Family 3 Subfamily A Member 4
DAPI: 4',6-Diamidino-2-Phenylindole, Dihydrochloride
DMEM: Dulbecco’s minimum essential medium
EGF: Epidermal growth factor
ELISA: Enzyme linked immunosorbent assay
FBS: Fetal bovine serum
FGF10: Fibroblast Growth Factor-10
GAPDH: Glyceraldehyde-3-phosphate dehydrogenase
GCV: Ganciclovir
GDNF: Glial cell line-derived neurotrophic factor
GFRa1: Glycosylphosphatidylinositol (GPI)-linked GDNF family receptor1
HE: Hematoxylin-eosin
HepLPCs: Human hepatocytes derived liver progenitor-like cells
hESCs: Human embryonic stem cells
HGF: Hepatocyte growth factor
HNF4A: Hepatocyte Nuclear Factor 4 Alpha
HSV-TK: Herpes simplex virus type 1 thymidine kinase
ICG: Indocyanine green
IgG: Immunoglobulin G
IHC: Immunohistochemistry
IRES: Internal ribosomal entry site
KRT19: Cyto-keratin19
KRT7: Cyto-keratin7
LiGEP: Liver-specific gene expression panel
LSECs: Liver sinusoidal endothelial cells
MMPs: Matrix metallopeptidases
MRP: Multidrug resistance-associated protein
MSCs: Mesenchymal stem cells
NAFLD: Non-alcoholic fatty liver disease
PAS: Periodic Acid-Schiff
PBS: Phosphate buffered saline
PHH: Human primary hepatocytes
PSCs: Induced pluripotent stem cells
RET: Rearranged in transfection
RT-PCR: Reverse Transcription-Polymerase Chain Reaction
SV40: Simian virus 40
TEM: Transmission Electron Microscope
VEGF: Vascular endothelial growth factor
vHepLPCs: HepLPC + HUVECs (1:1 mix)
ZO1/2: Zonula occludens 1/2
