# Supplementary material for: Establishment of Functional Liver Spheroids From Human Hepatocyte-Derived Liver Progenitor-Like Cells for Cell Therapy
Source: Front Bioeng Biotechnol. 2021 Nov 8;9:738081. doi: 10.3389/fbioe.2021.738081 (PMC8630579; doi:10.3389/fbioe.2021.738081)

Supplement figure 1

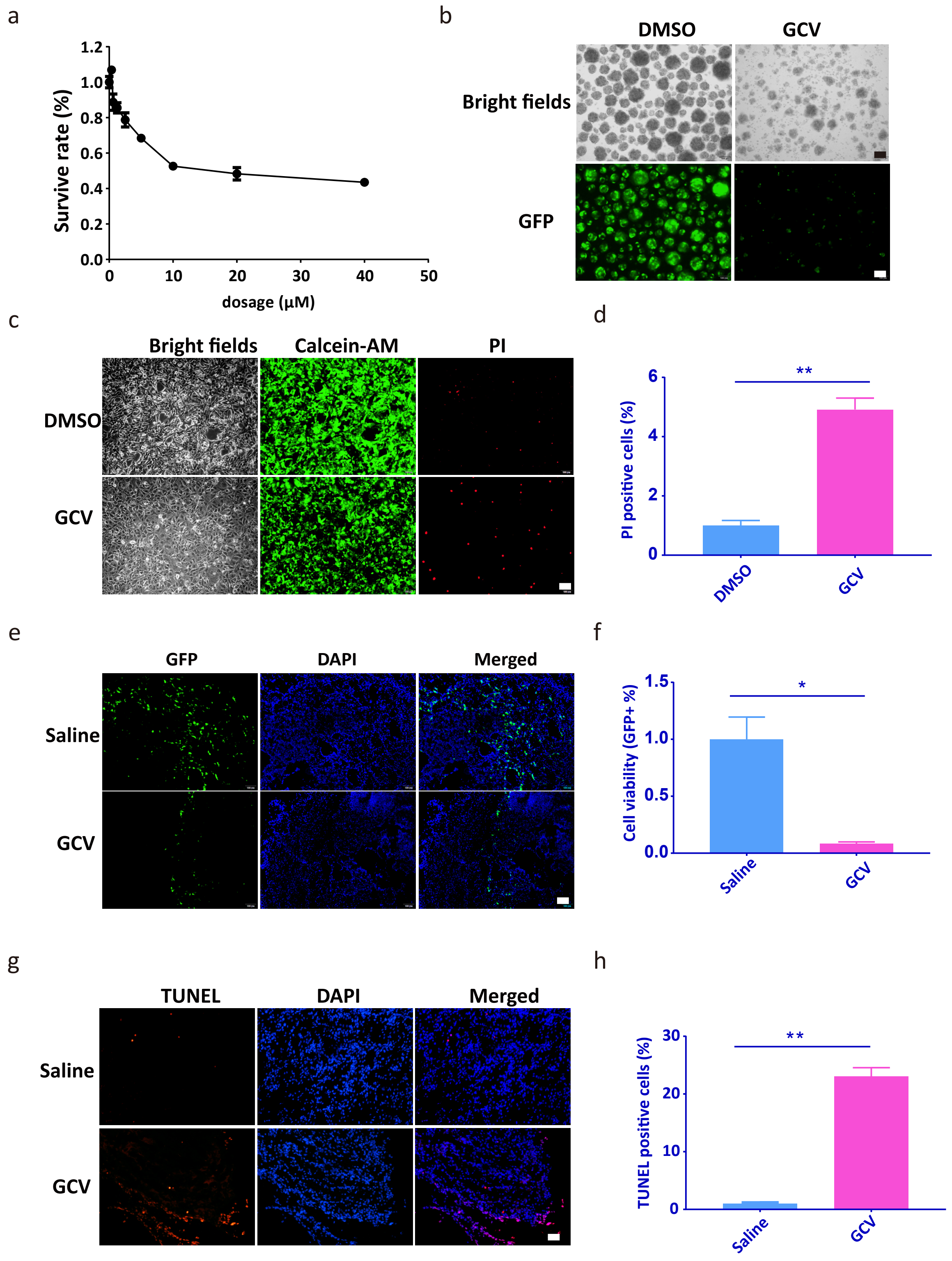

Supplement figure 2

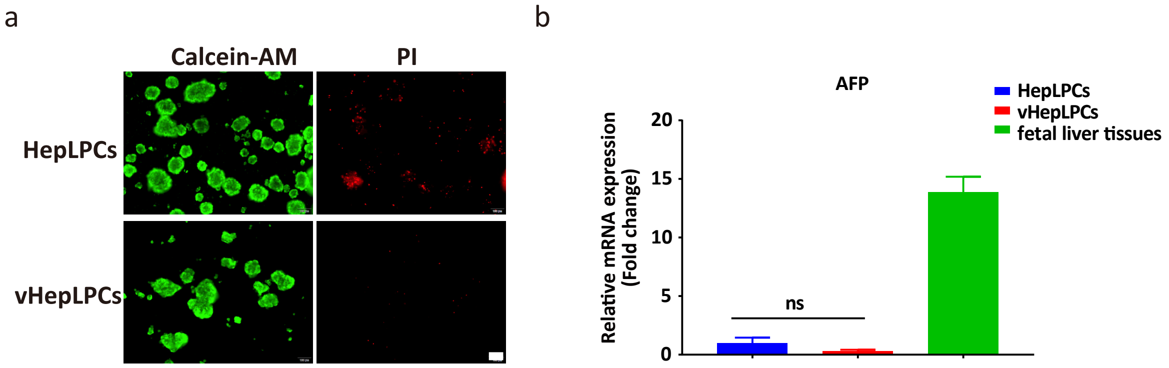

Supplement figure 3

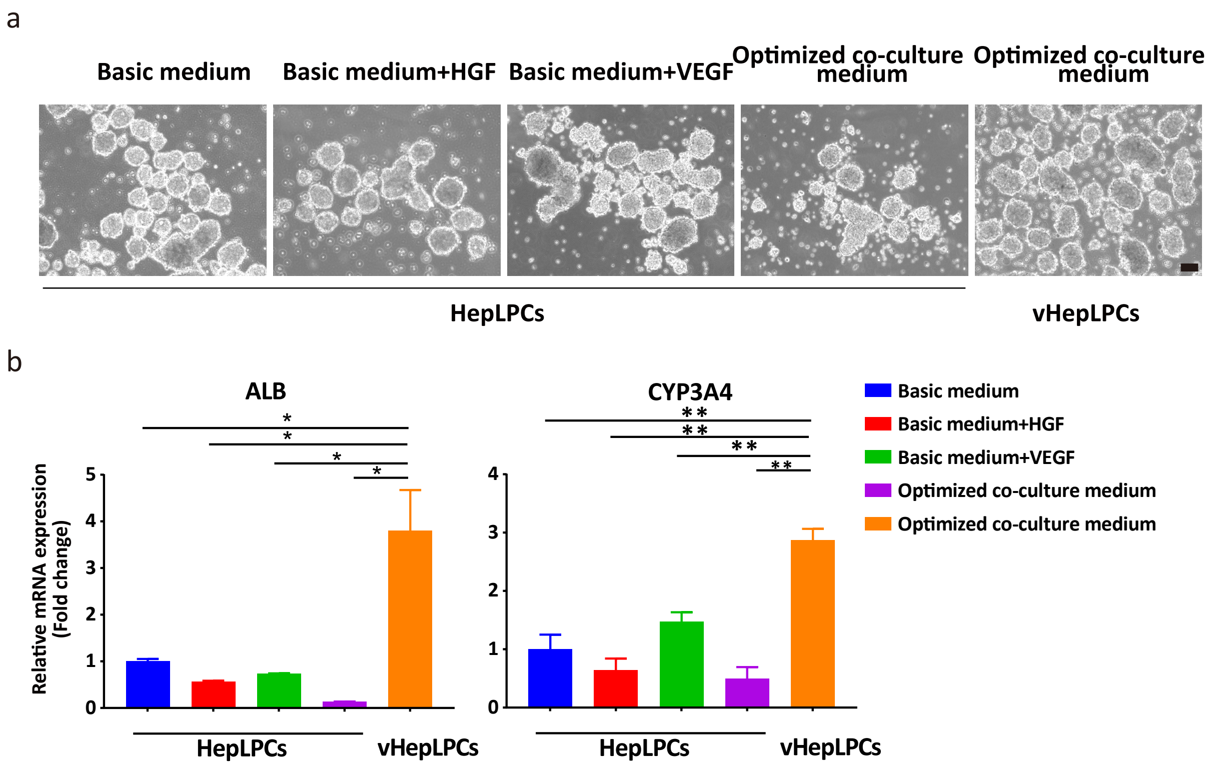

Supplement figure 4

a

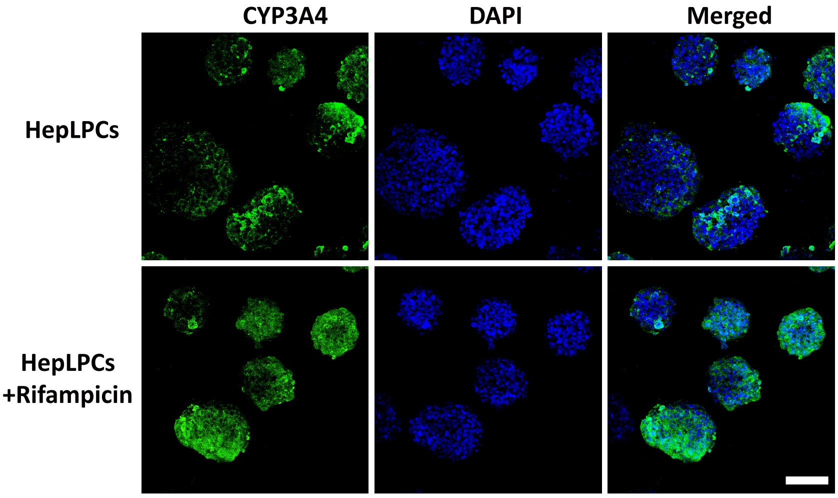

Supplement figure 5

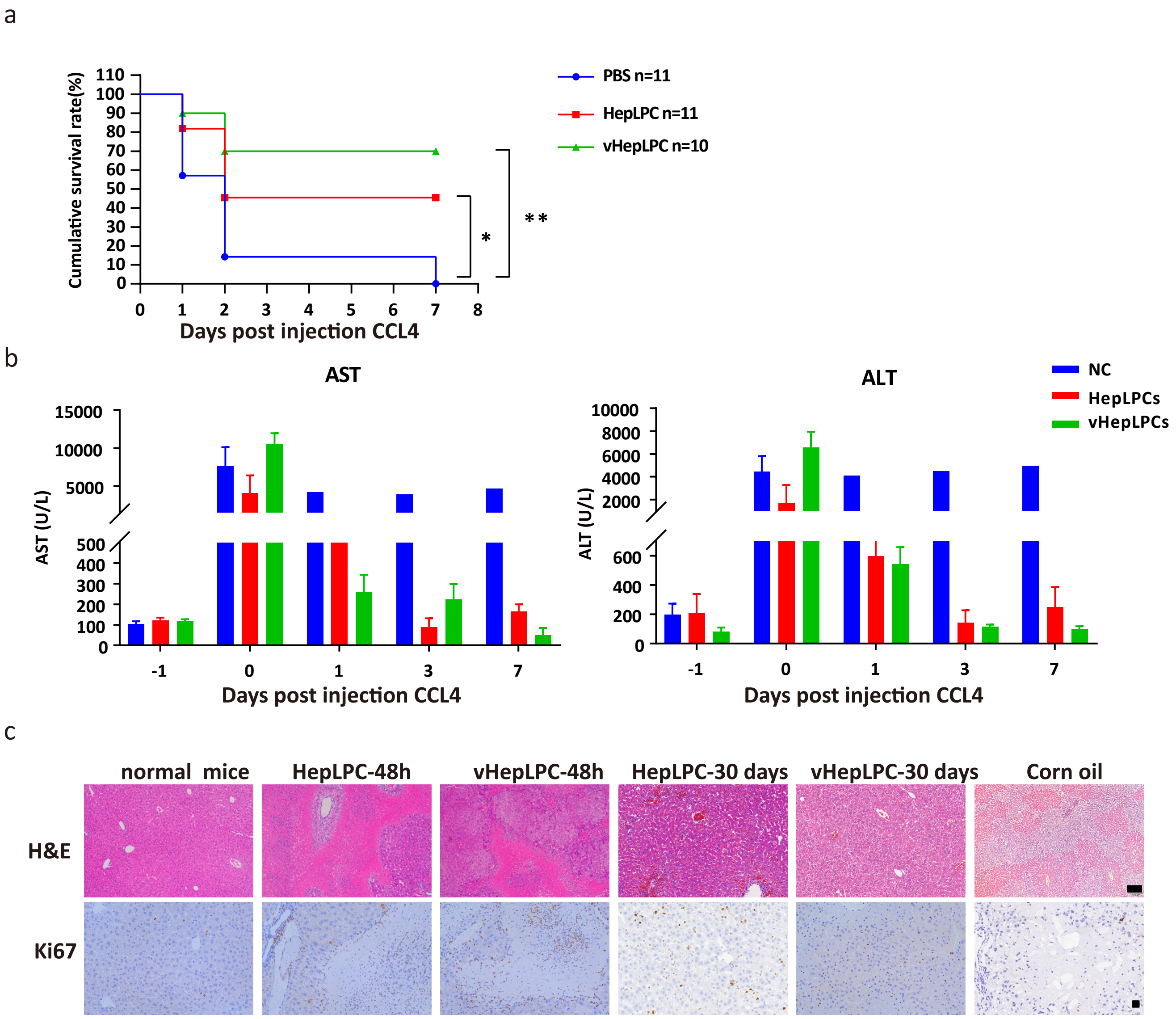

Supplement figure 6

a

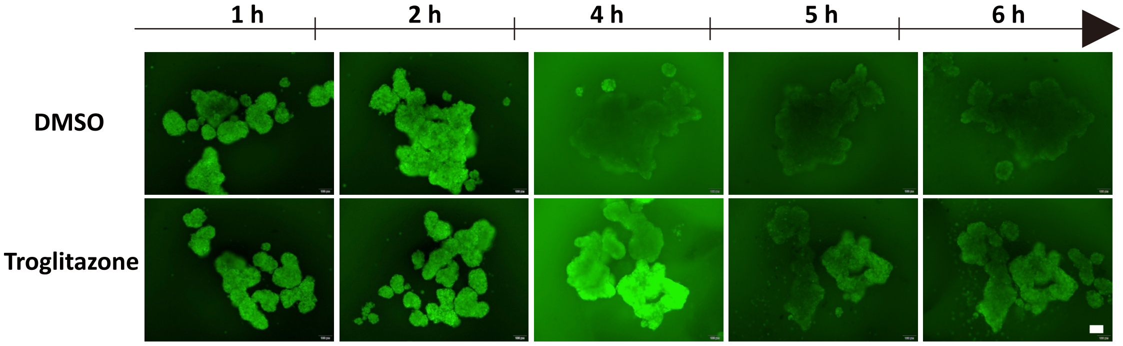

b

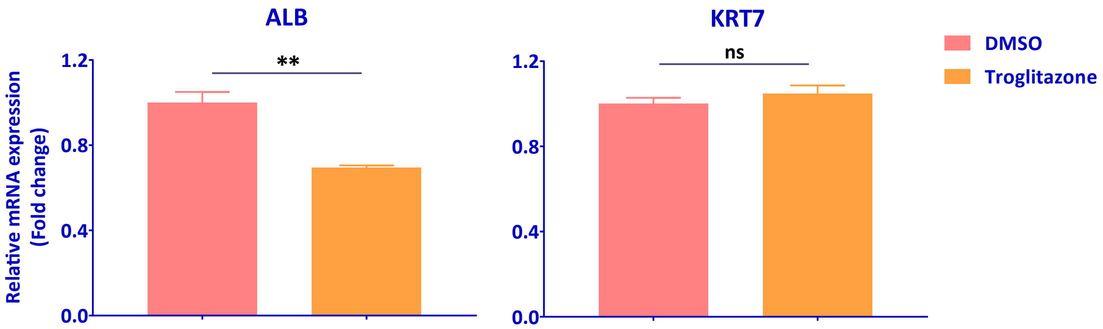

Supplement figure 7

a

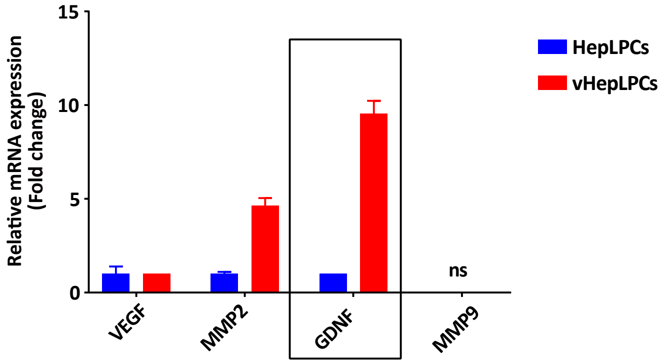

Supplement: Supplementary file 1 [file DataSheet1.PDF]
